# 2-(4-Pyridinio)benzimidazolium tetra­chloridopalladium(II)

**DOI:** 10.1107/S1600536808043523

**Published:** 2009-01-08

**Authors:** Jie Chen, Shao-Fan Wu

**Affiliations:** a Department of Civil Engineering, Fujian University of Technology, Fuzhou, Fujian 350002, People’s Republic of China; b Fujian Institute of Research on the Structure of Matter, Chinese Academy of Sciences, Fuzhou, Fujian 350002, People’s Republic of China

## Abstract

The asymmetric unit of the title compound, (C_12_H_11_N_3_)[PdCl_4_], consists of a 2-(4-pyridinio)benzimidazolium cation and two half [PdCl_4_]^2−^ anions, which are located on inversion centres. The cations lie in sheets parallel to (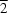
1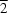). The cations and anions are connected by N—H⋯Cl and C—H⋯Cl contacts.

## Related literature

For related structures, see: Alcade *et al.* (1992 ▶); Chen *et al.* (2006 ▶); Huang *et al.* (2004 ▶); Wang *et al.* (1999 ▶).
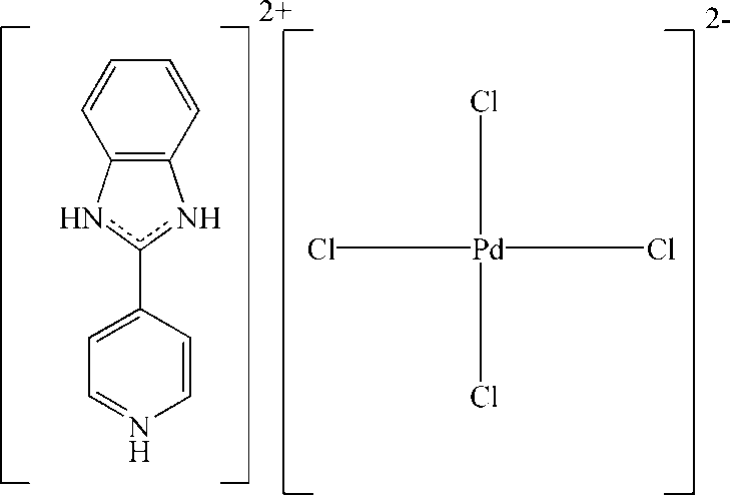


## Experimental

### 

#### Crystal data

(C_12_H_11_N_3_)[PdCl_4_]
*M* *_r_* = 445.44
Triclinic, 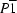

*a* = 8.2221 (1) Å
*b* = 8.3964 (2) Å
*c* = 12.3768 (5) Å
α = 94.09 (3)°
β = 97.42 (2)°
γ = 116.102 (10)°
*V* = 752.95 (11) Å^3^
*Z* = 2
Mo *K*α radiation
μ = 1.93 mm^−1^
*T* = 293 (2) K
0.30 × 0.15 × 0.04 mm

#### Data collection

Rigaku Mercury CCD diffractometer
Absorption correction: multi-scan (*CrystalClear*; Rigaku, 2002 ▶) *T*_min_ = 0.713, *T*_max_ = 0.916
5851 measured reflections
3406 independent reflections
2816 reflections with *I* > 2σ(*I*)
*R*_int_ = 0.017

#### Refinement

*R*[*F* ^2^ > 2σ(*F* ^2^)] = 0.030
*wR*(*F* ^2^) = 0.074
*S* = 1.06
3406 reflections
184 parameters
H-atom parameters constrained
Δρ_max_ = 0.81 e Å^−3^
Δρ_min_ = −0.61 e Å^−3^

### 

Data collection: *CrystalClear* (Rigaku, 2002 ▶); cell refinement: *CrystalClear*; data reduction: *CrystalClear*; program(s) used to solve structure: *SHELXS97* (Sheldrick, 2008 ▶); program(s) used to refine structure: *SHELXL97* (Sheldrick, 2008 ▶); molecular graphics: *DIAMOND* (Brandenburg & Putz, 2006 ▶) and *SHELXTL* (Sheldrick, 2008 ▶); software used to prepare material for publication: *SHELXL97*.

## Supplementary Material

Crystal structure: contains datablocks I, global. DOI: 10.1107/S1600536808043523/bt2834sup1.cif

Structure factors: contains datablocks I. DOI: 10.1107/S1600536808043523/bt2834Isup2.hkl

Additional supplementary materials:  crystallographic information; 3D view; checkCIF report

## Figures and Tables

**Table 1:** Hydrogen-bond geometry (Å, °)

*D*—H⋯*A*	*D*—H	H⋯*A*	*D*⋯*A*	*D*—H⋯*A*
N1—H1*A*⋯Cl4	0.86	2.55	3.207 (3)	134
N2—H2*A*⋯Cl3^i^	0.86	2.32	3.165 (2)	167
N3—H3*A*⋯Cl1	0.86	2.28	3.138 (3)	172
C5—H5*A*⋯Cl1^ii^	0.93	2.64	3.556 (3)	167

Symmetry codes: (i) 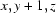; (ii) 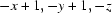.

**Fig. 1.**
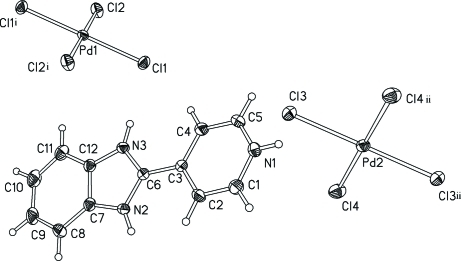
The structure of the title compound with the atomic labels and 30% probability displacement ellipsoids for non-H atoms. Symmetry codes: (i) 2 - x, 2 - y, -z; (ii) -x, -y, 1 - z.

**Fig. 2.**
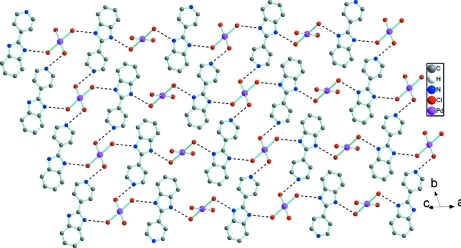
Hydrogen bond pattern of (I). The H atoms have been omitted for clarity; hydrogen bonds are shown as dashed lines.

**Fig. 3.**
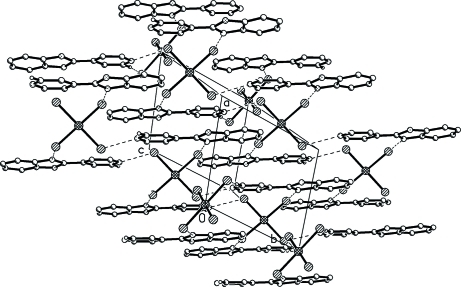
The crystal packing of (I), the N—H···Cl interactions are shown as dashed lines.

## supplementary crystallographic information

### Comment

The 2-(4-pyridyl)benzimidazole ligand is often used to act as terminal or bridging ligand in complexes, the noncoordinating N—H and N groups act as hydrogen bond donor or acceptor for the formation of hydrogen bonds, contributing to the crystal packing. Herein we report the synthesis and structure of title complex.

The asymmetry unit of the crystal structure of the title compound comprises one protonated 2-(4-pyridinio)benzimidazolium cation and two independent half [PdCl_4_]^2-^ anion. Each Pd^II^ atom has  a slightly distorted square planar coordination geometry (Fig. 1). The N—H···Cl interactions generate a two-dimensional sheet structure, as show in Fig. 2. The sheets are further connected into a three-dimensional network *via* C—H···Cl contacts  (Fig. 3).

### Experimental

An aqueous solution of PdCl_2_ 10 ml (0.108 g, 0.61 mmol), 2-(4-pyridyl)benzimidazole (Alcade *et al.*, 1992) (0.12 g, 0.61 mmol) and concentrated HCl (5 ml) was stirred continuously for about 30 min. the solution was allowed to stand at room temperature for several days and produced red crystals of the title compound (yield 85%).

### Refinement

After checking their presence in the different map, all H atoms were fixed geometrically and allowed to ride on their parent atoms, with C—H = 0.93 Å, N—H = 0.86 Å, *U*iso(H)=1.2*U*eq(C,N).

### Crystal data

**Table:**

(C_12_H_11_N_3_)[PdCl_4_]	*Z* = 2
*M**_r_* = 445.44	*F*(000) = 436
Triclinic, *P*1	*D*_x_ = 1.964 Mg m^−^^3^
Hall symbol: -P 1	Mo *K*α radiation, λ = 0.71073 Å
*a* = 8.2221 (1) Å	Cell parameters from 1904 reflections
*b* = 8.3964 (2) Å	θ = 3.3–27.5°
*c* = 12.3768 (5) Å	µ = 1.93 mm^−^^1^
α = 94.09 (3)°	*T* = 293 K
β = 97.42 (2)°	Prism, red
γ = 116.102 (10)°	0.30 × 0.15 × 0.04 mm
*V* = 752.95 (11) Å^3^	

### Data collection

**Table:**

Rigaku Mercury CCD diffractometer	3406 independent reflections
Radiation source: fine-focus sealed tube	2816 reflections with *I* > 2σ(*I*)
graphite	*R*_int_ = 0.017
ω scans	θ_max_ = 27.5°, θ_min_ = 3.3°
Absorption correction: multi-scan (*CrystalClear*; Rigaku, 2002)	*h* = −10→10
*T*_min_ = 0.713, *T*_max_ = 0.916	*k* = −10→10
5851 measured reflections	*l* = −16→15

### Refinement

**Table:**

Refinement on *F*^2^	Primary atom site location: structure-invariant direct methods
Least-squares matrix: full	Secondary atom site location: difference Fourier map
*R*[*F*^2^ > 2σ(*F*^2^)] = 0.030	Hydrogen site location: inferred from neighbouring sites
*wR*(*F*^2^) = 0.074	H-atom parameters constrained
*S* = 1.06	*w* = 1/[σ^2^(*F*_o_^2^) + (0.0306*P*)^2^ + 0.584*P*] where *P* = (*F*_o_^2^ + 2*F*_c_^2^)/3
3406 reflections	(Δ/σ)_max_ < 0.001
184 parameters	Δρ_max_ = 0.81 e Å^−^^3^
0 restraints	Δρ_min_ = −0.61 e Å^−^^3^

### Special details

**Table:**

Geometry. All e.s.d.'s (except the e.s.d. in the dihedral angle between two l.s. planes) are estimated using the full covariance matrix. The cell e.s.d.'s are taken into account individually in the estimation of e.s.d.'s in distances, angles and torsion angles; correlations between e.s.d.'s in cell parameters are only used when they are defined by crystal symmetry. An approximate (isotropic) treatment of cell e.s.d.'s is used for estimating e.s.d.'s involving l.s. planes.
Refinement. Refinement of *F*^2^ against ALL reflections. The weighted *R*-factor *wR* and goodness of fit *S* are based on *F*^2^, conventional *R*-factors *R* are based on *F*, with *F* set to zero for negative *F*^2^. The threshold expression of *F*^2^ > σ(*F*^2^) is used only for calculating *R*-factors(gt) *etc*. and is not relevant to the choice of reflections for refinement. *R*-factors based on *F*^2^ are statistically about twice as large as those based on *F*, and *R*- factors based on ALL data will be even larger.

### Fractional atomic coordinates and isotropic or equivalent isotropic displacement parameters (Å^2^)

**Table:**

	*x*	*y*	*z*	*U*_iso_*/*U*_eq_	
Pd1	1.0000	1.0000	0.0000	0.02528 (9)	
Pd2	0.0000	0.0000	0.5000	0.02595 (9)	
C1	0.2054 (5)	0.5925 (5)	0.3660 (3)	0.0415 (8)	
H1B	0.1425	0.5664	0.4248	0.050*	
C2	0.2983 (5)	0.7664 (4)	0.3483 (3)	0.0367 (7)	
H2B	0.2983	0.8586	0.3946	0.044*	
C3	0.3925 (4)	0.8040 (4)	0.2610 (2)	0.0281 (6)	
C4	0.3862 (5)	0.6625 (4)	0.1924 (3)	0.0389 (8)	
H4A	0.4468	0.6845	0.1325	0.047*	
C5	0.2904 (5)	0.4902 (4)	0.2129 (3)	0.0390 (8)	
H5A	0.2849	0.3947	0.1669	0.047*	
C6	0.4975 (4)	0.9893 (4)	0.2438 (2)	0.0269 (6)	
C7	0.6195 (4)	1.2852 (4)	0.2627 (3)	0.0318 (7)	
C8	0.6740 (5)	1.4663 (4)	0.2955 (3)	0.0428 (8)	
H8A	0.6320	1.5050	0.3532	0.051*	
C9	0.7928 (5)	1.5835 (5)	0.2379 (4)	0.0519 (10)	
H9A	0.8324	1.7056	0.2571	0.062*	
C10	0.8567 (5)	1.5272 (5)	0.1519 (4)	0.0523 (10)	
H10A	0.9340	1.6124	0.1137	0.063*	
C11	0.8097 (5)	1.3500 (5)	0.1212 (3)	0.0428 (8)	
H11A	0.8555	1.3130	0.0649	0.051*	
C12	0.6896 (4)	1.2291 (4)	0.1793 (3)	0.0326 (7)	
N1	0.2050 (4)	0.4609 (4)	0.2996 (2)	0.0376 (6)	
H1A	0.1475	0.3527	0.3128	0.045*	
N2	0.5007 (3)	1.1324 (3)	0.2995 (2)	0.0299 (6)	
H2A	0.4383	1.1300	0.3505	0.036*	
N3	0.6107 (4)	1.0441 (3)	0.1709 (2)	0.0305 (6)	
H3A	0.6313	0.9751	0.1258	0.037*	
Cl1	0.69047 (11)	0.81997 (12)	−0.00991 (7)	0.0429 (2)	
Cl2	0.95830 (12)	0.96403 (11)	−0.18905 (6)	0.0393 (2)	
Cl3	0.30176 (11)	0.19239 (11)	0.48955 (7)	0.03794 (19)	
Cl4	−0.09172 (12)	0.20039 (11)	0.43218 (7)	0.0396 (2)	

### Atomic displacement parameters (Å^2^)

**Table:**

	*U*^11^	*U*^22^	*U*^33^	*U*^12^	*U*^13^	*U*^23^
Pd1	0.02766 (18)	0.02197 (16)	0.02590 (17)	0.01006 (13)	0.00961 (13)	0.00102 (12)
Pd2	0.02897 (18)	0.02667 (17)	0.02514 (17)	0.01380 (14)	0.01011 (13)	0.00409 (13)
C1	0.046 (2)	0.0370 (18)	0.0417 (19)	0.0152 (16)	0.0212 (16)	0.0093 (15)
C2	0.044 (2)	0.0273 (16)	0.0375 (18)	0.0135 (15)	0.0144 (15)	0.0000 (13)
C3	0.0252 (15)	0.0271 (15)	0.0322 (16)	0.0113 (12)	0.0079 (12)	0.0042 (12)
C4	0.041 (2)	0.0327 (17)	0.0446 (19)	0.0152 (15)	0.0205 (16)	0.0054 (15)
C5	0.044 (2)	0.0293 (16)	0.045 (2)	0.0169 (15)	0.0129 (16)	−0.0019 (14)
C6	0.0248 (14)	0.0272 (14)	0.0292 (15)	0.0119 (12)	0.0059 (12)	0.0036 (12)
C7	0.0278 (16)	0.0287 (15)	0.0382 (17)	0.0123 (13)	0.0045 (13)	0.0077 (13)
C8	0.042 (2)	0.0283 (17)	0.059 (2)	0.0178 (15)	0.0087 (17)	0.0022 (16)
C9	0.041 (2)	0.0237 (16)	0.084 (3)	0.0088 (15)	0.008 (2)	0.0118 (18)
C10	0.043 (2)	0.041 (2)	0.071 (3)	0.0129 (18)	0.016 (2)	0.027 (2)
C11	0.0372 (19)	0.045 (2)	0.047 (2)	0.0156 (16)	0.0148 (16)	0.0177 (17)
C12	0.0295 (17)	0.0289 (15)	0.0392 (17)	0.0125 (13)	0.0065 (14)	0.0077 (13)
N1	0.0400 (16)	0.0259 (13)	0.0485 (17)	0.0138 (12)	0.0150 (13)	0.0101 (12)
N2	0.0311 (14)	0.0261 (13)	0.0356 (14)	0.0144 (11)	0.0118 (11)	0.0031 (11)
N3	0.0338 (14)	0.0278 (13)	0.0322 (14)	0.0140 (11)	0.0141 (11)	0.0045 (11)
Cl1	0.0280 (4)	0.0474 (5)	0.0406 (5)	0.0061 (4)	0.0134 (3)	−0.0099 (4)
Cl2	0.0485 (5)	0.0319 (4)	0.0271 (4)	0.0082 (4)	0.0105 (3)	0.0025 (3)
Cl3	0.0306 (4)	0.0347 (4)	0.0446 (5)	0.0098 (3)	0.0157 (4)	−0.0004 (3)
Cl4	0.0478 (5)	0.0428 (4)	0.0433 (5)	0.0293 (4)	0.0193 (4)	0.0180 (4)

### Geometric parameters (Å, °)

**Table:**

Pd1—Cl2	2.2987 (8)	C7—N2	1.380 (4)
Pd1—Cl1	2.2989 (8)	C7—C12	1.394 (4)
Pd2—Cl4	2.2937 (8)	C7—C8	1.395 (4)
Pd2—Cl3	2.3170 (8)	C8—C9	1.366 (5)
C1—N1	1.328 (4)	C8—H8A	0.9300
C1—C2	1.368 (4)	C9—C10	1.387 (6)
C1—H1B	0.9300	C9—H9A	0.9300
C2—C3	1.385 (4)	C10—C11	1.373 (5)
C2—H2B	0.9300	C10—H10A	0.9300
C3—C4	1.388 (4)	C11—C12	1.388 (4)
C3—C6	1.458 (4)	C11—H11A	0.9300
C4—C5	1.371 (5)	C12—N3	1.386 (4)
C4—H4A	0.9300	N1—Cl4	3.207 (3)
C5—N1	1.336 (4)	N1—H1A	0.8600
C5—Cl1^i^	3.556 (3)	N2—Cl3^ii^	3.165 (2)
C5—H5A	0.9300	N2—H2A	0.8600
C6—N2	1.330 (3)	N3—Cl1	3.138 (3)
C6—N3	1.338 (3)	N3—H3A	0.8600
			
Cl2—Pd1—Cl2^iii^	180.00 (4)	N2—C6—N3	108.6 (3)
Cl2—Pd1—Cl1	89.78 (4)	N2—C6—C3	125.9 (2)
Cl2^iii^—Pd1—Cl1	90.22 (4)	N3—C6—C3	125.5 (3)
Cl2—Pd1—Cl1	89.78 (4)	N2—C7—C12	106.6 (3)
Cl2^iii^—Pd1—Cl1	90.22 (4)	N2—C7—C8	131.9 (3)
Cl1—Pd1—Cl1	0.00 (4)	C12—C7—C8	121.5 (3)
Cl2—Pd1—Cl1^iii^	90.22 (4)	C9—C8—C7	116.0 (3)
Cl2^iii^—Pd1—Cl1^iii^	89.78 (4)	C9—C8—H8A	122.0
Cl1—Pd1—Cl1^iii^	180.0	C7—C8—H8A	122.0
Cl1—Pd1—Cl1^iii^	180.0	C8—C9—C10	122.4 (3)
Cl4—Pd2—Cl4	0.00 (7)	C8—C9—H9A	118.8
Cl4—Pd2—Cl4^iv^	180.0	C10—C9—H9A	118.8
Cl4—Pd2—Cl4^iv^	180.0	C11—C10—C9	122.2 (3)
Cl4—Pd2—Cl3^iv^	90.27 (3)	C11—C10—H10A	118.9
Cl4—Pd2—Cl3^iv^	90.27 (3)	C9—C10—H10A	118.9
Cl4^iv^—Pd2—Cl3^iv^	89.73 (3)	C10—C11—C12	116.1 (3)
Cl4—Pd2—Cl3	89.73 (3)	C10—C11—H11A	122.0
Cl4—Pd2—Cl3	89.73 (3)	C12—C11—H11A	122.0
Cl4^iv^—Pd2—Cl3	90.27 (3)	N3—C12—C11	132.4 (3)
Cl3^iv^—Pd2—Cl3	180.0	N3—C12—C7	105.9 (3)
N1—C1—C2	120.2 (3)	C11—C12—C7	121.7 (3)
N1—C1—H1B	119.9	C1—N1—C5	122.5 (3)
C2—C1—H1B	119.9	C1—N1—Cl4	85.01 (19)
C1—C2—C3	119.5 (3)	C5—N1—Cl4	151.2 (2)
C1—C2—H2B	120.3	C1—N1—H1A	118.7
C3—C2—H2B	120.3	C5—N1—H1A	118.7
C2—C3—C4	118.6 (3)	C6—N2—C7	109.5 (2)
C2—C3—C6	119.9 (3)	C6—N2—Cl3^ii^	134.59 (18)
C4—C3—C6	121.5 (3)	C7—N2—Cl3^ii^	115.88 (18)
C5—C4—C3	119.9 (3)	C6—N2—H2A	125.2
C5—C4—H4A	120.0	C7—N2—H2A	125.2
C3—C4—H4A	120.0	C6—N3—C12	109.4 (2)
N1—C5—C4	119.3 (3)	C6—N3—Cl1	130.00 (19)
N1—C5—Cl1^i^	129.9 (2)	C12—N3—Cl1	120.44 (18)
C4—C5—Cl1^i^	110.8 (2)	C6—N3—H3A	125.3
N1—C5—H5A	120.4	C12—N3—H3A	125.3
C4—C5—H5A	120.4		

Symmetry codes:  (i) −*x*+1, −*y*+1, −*z*; (ii) *x*, *y*+1, *z*; (iii) −*x*+2, −*y*+2, −*z*; (iv) −*x*, −*y*, −*z*+1.

### Hydrogen-bond geometry (Å, °)

**Table:**

*D*—H···*A*	*D*—H	H···*A*	*D*···*A*	*D*—H···*A*
N1—H1A···Cl4	0.86	2.55	3.207 (3)	134
N2—H2A···Cl3^ii^	0.86	2.32	3.165 (2)	167
N3—H3A···Cl1	0.86	2.28	3.138 (3)	172
C5—H5A···Cl1^i^	0.93	2.64	3.556 (3)	167

Symmetry codes:  (ii) *x*, *y*+1, *z*; (i) −*x*+1, −*y*+1, −*z*.
